# Low pathogenic avian influenza virus isolates with different levels of defective genome segments vary in pathogenicity and transmission efficiency

**DOI:** 10.1186/s13567-020-00833-6

**Published:** 2020-08-28

**Authors:** Edyta Świętoń, Karolina Tarasiuk, Krzysztof Śmietanka

**Affiliations:** Department of Poultry Diseases, National Veterinary Research Institute, Al. Partyzantów 57, 24-100 Puławy, Poland

**Keywords:** avian influenza, defective interfering particles, pathogenicity

## Abstract

Defective interfering particles (DIPs) of influenza virus are generated through incorporation of highly truncated forms of genome segments, mostly those coding polymerase complex proteins (PB2, PB1, PA). Such particles are able to replicate only in the presence of a virus with the complete genome, thus DIPs may alter the infection outcome by suppressing production of standard virus particles, but also by stimulating the immune response. In the present study we compared the clinical outcome, mortality and transmission in chickens and turkeys infected with the same infectious doses of H7N7 low pathogenic avian influenza virus containing different levels of defective gene segments (95/95(DVG-high) and 95/95(DVG-low)). No clinical signs, mortality or transmission were noted in SPF chickens inoculated with neither virus stock. Turkeys infected with 95/95(DVG-high) showed only slight clinical signs with no mortality, and the virus was transmitted only to birds in direct contact. In contrast, more severe disease, mortality and transmission to direct and indirect contact birds was observed in turkeys infected with 95/95(DVG-low). Apathy, lower water and food intake, respiratory system disorders and a total mortality of 60% were noted. Shedding patterns in contact turkeys indicated more efficient within- and between-host spread of the virus than in 95/95(DVG-high) group. Sequencing of virus genomes showed no mutations that could account for the observed differences in pathogenicity. The results suggest that the abundance of DIPs in the inoculum was the factor responsible for the mild course of infection and disrupted virus transmission.

## Introduction

The genome of influenza A virus consists of eight single-stranded negative-sense RNA segments numbered according to their length in a descending order [1]. The length of segments 1 and 2 is 2341 nucleotides (nt) and segment 3 contains 2233 nt. They encode proteins of the polymerase complex (polymerase basic 2, PB2; polymerase basic 1, PB1; polymerase acidic, PA, respectively). The mid-length segments 4–6 code for hemagglutinin (HA), nucleoprotein (NP) and neuraminidase (NA). The M (matrix) and NS (non-structural) segments are 1027 nt and 890 nt in length, respectively, and each codes for two proteins: M1 and M2, and NS1 and NEP [1]. The RNA segments are associated with PB2, PB1, PA and NP forming ribonucleoprotein complexes (vRNP), which are basic replication units [2]. Replication of influenza virus is an error-prone process but apart from point mutations introduced during the synthesis of novel RNA molecules, defective viral segments are also produced and assembled into virus particles [3]. Virions containing highly deleted forms of genome segments (defective viral genes—DVGs) are able to replicate only in the presence and at the expense of fully infectious virus, hence the term “defective interfering particles” (DIPs) [4]. Generation of DIPs was first described in 1940s by von Magnus, who noted that passages of non-diluted influenza virus in embryonated eggs led to a gradual decrease of the infectious titre despite an increase in the amount of viral particles [5]. Further studies showed that this phenomenon is common for RNA and DNA viruses passaged at high multiplicity of infection in laboratory conditions, but DIPs were found also in natural infections [6–9]. Defective genomes of influenza virus arise through deletion of a large portion in the middle part of genome segments, mostly in PB2, PB1 and PA, while the 5′ and 3′ termini with packaging signals are retained [10]. Therefore, in cells co-infected with both defective and standard virus particles, the production and packaging of shortened genome segments outcompetes those of full-length [11].

Due to the ability of in vitro derived DIPs to interfere with replication of full virus particles and induce immune response, their potential application as antiviral and immunostimulatory agents has been studied extensively in recent years [12–14]. However, the biological role of DIPs emerging during in vivo infection is not clearly defined. They might be a tolerable effect of rapid, but error-prone replication [15]. Otherwise, they might mitigate the disease enabling survival of the host thus favouring virus spread [12]. Several studies on influenza in humans and animals suggest the latter possibility is more likely [16–18]. Experiments on the effect of influenza DIPs in mice showed that protection from severe disease is a result of reduction of the amount of infectious virus [19] or modulation of host immune response [20, 21]. The DIP-mediated stimulation of innate immunity occurs due to a preferential recognition of short viral RNAs by retinoic acid inducible gene I (RIG-I), one of cellular sensor of viral RNA, whose activation initiates antiviral and inflammatory response [22]. Despite numerous evidence that the DIPs activity may reduce the disease severity and increase host survivability in mice and ferrets, there is little data on their properties during infection with avian influenza in birds. It was demonstrated that increased generation of defective particles might had contributed to the reduced virulence of a highly pathogenic H5N2 avian influenza virus in chickens [16, 23]. To evaluate the effect of DIPs on the course of infection with low pathogenic avian influenza virus (LPAIV), a comparison of pathogenicity of two virus stocks of H7N7 LPAIV with different levels of defective genomes was performed in turkeys and chickens.

## Materials and methods

### Virus stocks

The low pathogenic avian influenza virus A/turkey/Poland/95/1995(H7N7) was used. This strain represented a group of LPAIV H7N7 causing outbreaks in meat and breeder turkeys in Poland in mid-1990s [24]. Despite low pathogenicity of the virus in experimental chickens (intravenous pathogenicity index, IVPI = 0.0), clinical disease was observed in the field outbreaks. Respiratory symptoms were noted in meat turkeys and breeders showed also a drop in egg production [24].

The virus stock from the 7^th^ passage in embryonated specific pathogen free (SPF) chicken eggs was shown to have contained a high level of defective viral gene segments (see below), therefore it was designated as 95/95(DVG-high). To reduce the amount of defective particles from the virus stock, additional three passages were performed in SPF chicken eggs using highly diluted inoculum (10^–6^) and short incubation periods (24–45 h). The resulting virus stock was designated as 95/95(DVG-low). Both isolates were characterized by hemagglutination (HA) assay and titrated in SPF chicken eggs according to standard procedures [25].

### Whole-genome sequencing

The RNA was extracted from 95/95(DVG-high) and 95/95(DVG-low) stocks using Viral Mini Kit (Syngen, Poland) following the manufacturer’s instructions. The virus genome was amplified in RT-PCR using universal primers flanking all eight genome segments [26] with a modification to improve the yield of PB2, PB1 and PA segments [27]. Reactions were performed with SuperScript III One-Step RT-PCR System with Platinum Taq High Fidelity DNA Polymerase (ThermoFisher Scientific, USA). The PCR products were purified using PCR/DNA Clean-Up Purification Kit (EURx, Poland) and processed with Nextera XT DNA Library Preparation Kit (Illumina, USA) according to the manufacturer’s manual. The obtained libraries were sequenced in MiSeq using paired-end 300 bp mode (Illumina. USA). Raw reads were cleaned using Trimmomatic [28] and aligned using BWA [29] and a sequence of a wild-bird origin H7N7 AIV as the reference genome. Consensus sequences were generated with Samtools [30] and reads were mapped again using these sequences as references. Coverage data were obtained using Samtools. Consensus sequences of 95/95(DVG-high) and 95/95(DVG-low) obtained after the second round of mapping were compared to identify any mutations that may result in an altered pathogenicity. In addition, variant calling was performed using VarScan [31] and variants of ≥ 5% were included in further analysis.

### Analysis of amount of defective segments

To assess the differences in the proportions of defective and full genome segments, a real time RT-PCR method was developed with two sets of primers and probe targeting different regions of the PB2 and PA genes (Table 1). The primer and probe set binding near the 3′ terminus (T) of the gene allows detection of both defective and full segments, while the set targeting the internal (I) part of segment allows detection of those of full length only. Reactions were performed using QuantiTect Probe RT-PCR Kit (Qiagen, Germany) according to the manufacturer’s recommendations in an ABI 7500 Fast System. Three biological replicates were tested for each virus stock. Based on the Ct values obtained with terminal and internal assays, relative differences in the amount of defective RNAs between 95/95(DVG-high) and 95/95(DVG-low) were evaluated as previously described [32]. Briefly, a ratio 2^(-CtT)^:2^(-CtI)^ was calculated and compared for both virus stocks. Welch t-test was used to assess whether the differences were statistically significant with *p* value of 0.05 as a threshold.

**Table 1:** **Primer and probe sequences designed to distinguish between the defective and full viral segments in 95/95(DVG-high) and 95/95(DVG-low) virus isolates**

Location	Name of primer/probe	Sequence 5′–3’
Terminal PB2	PB2-beg-F	GTCAAATATATTCAATATGGAGAG
	PB2-beg-R	GTGGTCTTTGTTAGTATCTCG
	PB2-beg-pro	[FAM]-AAGAGATTTGATGTCGCAGTCTC-[TAM]
Internal PB2	PB2-mid-F	AAGAGCAACAGCCATTCTAA
	PB2-mid-R	ATCGCTACTATGATCGCTTC
	PB2-mid-pro	[FAM]-CAGAAGGCTGATTCAACTGATAG-[TAM]
Terminal PA	PA-beg-F	ATGGAAGACTTTGTGCGACAA
	PA-beg-R	AAACAGACTTCTAAGTGTGTGC
	PA-beg-pro	[FAM]-AATGATTGTCGAGCTTGCGGAAAAG-[TAM]
Internal PA	PA-mid-F	AGTGGGCACTTGGTGAGA
	PA-mid-R	ATCCAGCTTGCTAGCGATC
	PA-mid-pro	[FAM]-TTCATCACTGTCATACTGTCTTAGAT-[TAM]

### Cloning and sequencing of defective segments

The defective segments were amplified with the eight-segment protocol as described above. The PCR products were separated in 2% agarose and a band of 400–600 bp was excised from the gel and extracted using NucleoSpin Gel and PCR Clean-up kit (Macherey Nagel, Germany) according to the manufacturer’s protocol. The purified fragments were cloned into a plasmid using TOPO TA Cloning Kit for Sequencing (ThermoFisher Scientific, USA) as per the manufacturer’s manual. Colonies containing plasmids with inserts were identified by colony PCR. Twenty clones were sequenced using M13 primers and BigDye Terminator v3.1 Cycle Sequencing Kit (ThermoFisher Scientific, USA) in 3500 Genetic Analyzer. Sequences of inserts were assembled and analyzed in SeqScape v2.7 (Applied Biosystems, USA).

### Animal experiments

The experiments were performed in 3-week-old SPF chickens and commercial turkeys. Chickens were hatched from SPF eggs (VALO BioMedia, Germany) and 1-day-old turkey poults were purchased from a commercial hatchery. Birds were reared until the age of 3 weeks in the animal facility of the National Veterinary Research Institute (NVRI), Poland. Experiments for each virus stock and each species were conducted separately in the BSL3 + animal facility. Birds were housed in open metal grid cages with feed and water provided ad libitum. To exclude previous exposure to AIV, blood samples and swabs were collected before infection and tested by serological and molecular methods, respectively. Additionally, turkeys were examined for the presence of common viruses and bacteria that may exacerbate infection with AIV: *Mycoplasma* spp., *Ornithobacterium rhinotracheale*, turkey coronavirus, astrovirus, rotavirus, adenovirus, parvovirus, avian metapneumovirus, and reovirus (protocols available upon request) with negative results. Birds were randomly divided into three groups: inoculated, direct contact (placed in the same cage) and indirect contact (placed in a neighbouring cage at a distance of approximately 50 cm). Five birds were inoculated intranasally and intraocularly with the dose of 10^6^ EID_50_ of either 95/95(DVG-high) or 95/95(DVG-low) in a total volume of 0.1 mL. At 1 day post infection (dpi) five direct contact birds were placed in the same cage and another five birds were placed in an adjacent cage to monitor the indirect contact transmission of the virus. Birds were monitored daily for the presence of clinical signs and mortality. At 1, 3, 5, 7, 10 and 14 dpi oropharyngeal and cloacal swabs were collected and immersed in viral transport medium (Copan, Italy). At 14 dpi blood samples were collected and birds were humanely euthanized.

### Assessment of virus shedding and seroconversion

The RNA was extracted from 200 µL of swab sample using Viral RNA Mini Kit (Syngen, Poland). The viral load was examined without discrimination between defective and standard genomes using primers and a probe targeting the M gene [33] with QuantiTect Probe RT-PCR Kit (Qiagen, Germany). Ten-fold dilutions of the virus inoculum were used to generate standard curve and calculate the equivalents of EID_50_ (eqEID_50_) per 0.1 mL of swab medium. Statistical analysis for the corresponding 95/95(DVG-high) and 95/95(DVG-low) groups was performed using Mann–Whitney test with *p* < 0.05 considered statistically significant. Due to the mortality in 95/95(DVG-low) groups, comparisons were made for 1, 3, 5 and 7 dpi in the case of inoculated birds, 3, 5 and 7 dpi in the case of direct contact birds, and 3, 5, 7, 10 for indirect contact birds. Serum samples were tested in hemagglutination inhibition (HI) test using homologous H7N7 antigen according to a standard procedure [25].

### Analysis of virus genomes in swabs

The AIV-positive RNAs from oropharyngeal swabs collected at 5 dpi were subjected to whole genome amplification and deep sequencing as described above. Additionally, sequencing of HA cleavage site (HACS) for swabs from 10 and 14 dpi, and a kidney sample collected from euthanized bird at 14 dpi was performed. Briefly, a fragment encompassing the HACS was amplified in RT-PCR using GK7.3 and GK7.4 primers [34] with OneStep RT-PCR Kit (Qiagen, Germany). The PCR products were sequenced using BigDye Terminator v3.1 Cycle Sequencing Kit in 3500 Genetic Analyzer (Applied Biosystems, USA). The sequences were analyzed in SeqScape v2.7 (Applied Biosystems, USA).

## Results

### Characterization of 95/95(DVG-high) and 95/95(DVG-low) virus stocks

**Figure 1:**
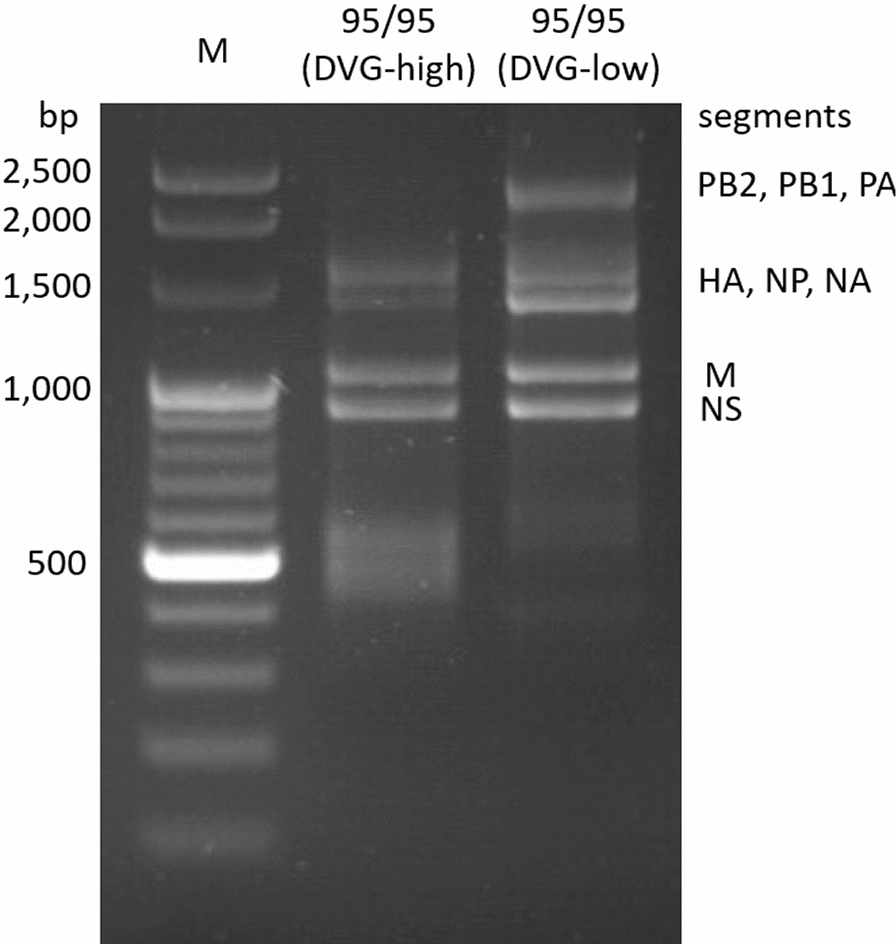
**Electrophoresis of PCR products obtained with eight-segment amplification protocol performed for 95/95(DVG-high) and 95/95(DVG-low) virus stocks.** The size of bands of molecular marker (M) is shown on the left and positions of amplification products of full length genome segments are indicated on the right of the gel.

**Figure 2:**
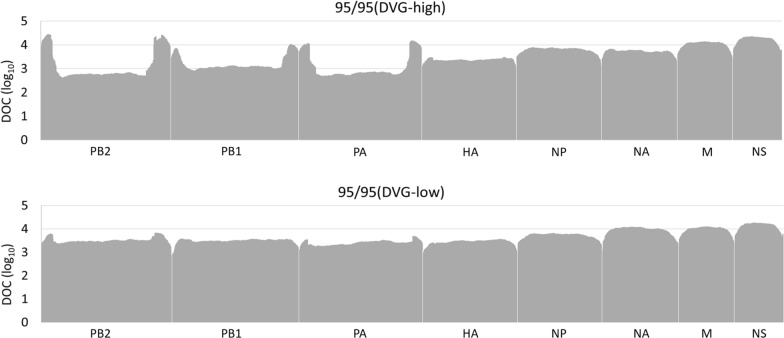
**Coverage for genome segments of 95/95(DVG-high) and 95/95(DVG-low) virus stocks obtained in deep sequencing.** The X-axis represent the depth of coverage (DOC) an the Y-axis corresponds to the genomic position across each genome segment.

Titration of 95/95(DVG-high) and 95/95(DVG-low) showed an increase in the amount of infectious virus (from 10^6.63^ EID_50_/0.1 mL to 10^8.38^ EID50/0.1 mL, respectively), despite similar quantity of virus particles as evidenced by identical HA titre. The presence of DVGs in 95/95(DVG-high) was confirmed in RT-PCR by poor amplification of long genome segments and presence of short PCR products of about 400–600 bp (Figure 1). Coverage plots obtained from deep sequencing data revealed uneven distribution of reads mapped to the PB2, PB1 and PA segments with high coverage in the 3′ and 5′ termini (Figure 2). The highest disproportion between the segment ends and internal part was observed for the PB2 gene. In the case of 95/95(DVG-low), efficient amplification of long segments was noted (Figure 1). Weak bands indicative of shortened segments were also observed, but with a different length pattern than that in 95/95(DVG-high) (Figure 1). Deep sequencing confirmed the low level of defective segments in 95/95(DVG-low), noticeable only in the case of PB2 and PA genes (Figure 2). The differences in the amount of truncated forms were also identified in real time RT-PCR targeting distinct fragments of the PB2 and PA genes (Table 2). Higher 2^(−CtT)^:2^(−CtI)^ ratios were found for 95/95(DVG-high) (*p* < 0.05 for PB2 and PA) indicating higher levels of DVGs than in 95/95(DVG-low).

**Table 2:** **Results of real time RT-PCR targeting terminal and internal fragments of PB2 and PA segments in 95/95(DVG-high) and 95/95(DVG-low) virus isolates. Ratios of 2**^(−Ct)^** for terminal and internal assay are presented with standard deviation of three replicates in parenthesis**

Genome segment	2^(−CtT)^:2^(−CtI)^ ratio	
	95/95(DVG-high)	95/95(DVG-low)
PB2	16.59 (2.88)	2.96 (0.08)
PA	4.02 (1.00)	1.84 (0.2)

**Figure 3:**
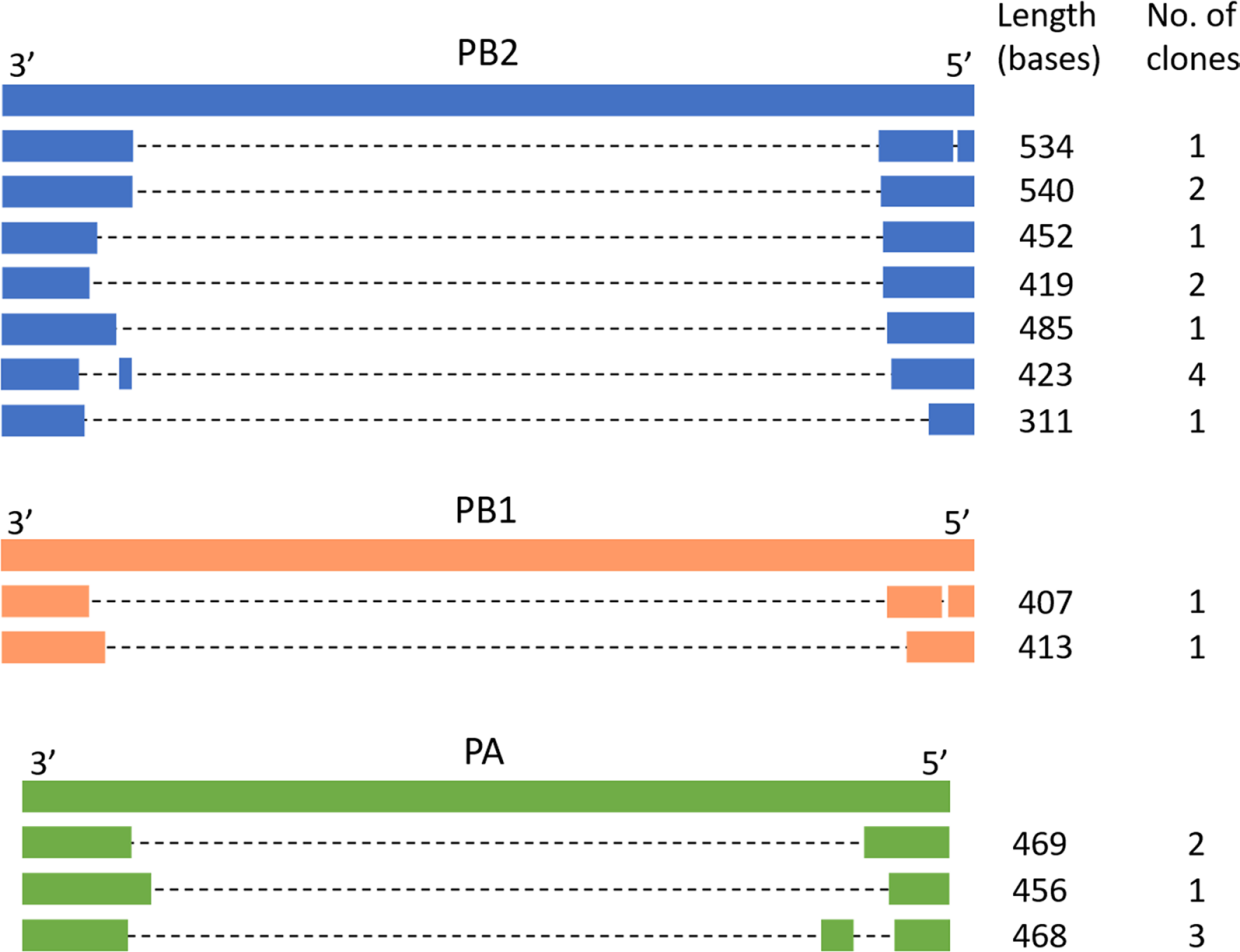
**Patterns of deletions found in defective forms of PB2, PB1 and PA segments of the 95/95(DVG-high).**

Sequencing of defective segments of 95/95(DVG-high) cloned into plasmids showed that all 20 clones contained defective forms of polymerase complex genes. Several patterns of deletions were found for each segment (Figure 3). The highest number of clones contained defective forms of the PB2 gene which showed also the highest diversity in terms of length and position of deletions (Figure 3).

Full-length sequences of all gene segments obtained in deep sequencing were compared to identify mutations that may affect the virus pathogenicity. Four consensus-level nonsynonymous differences between 95/95(DVG-low) and 95/95(DVG-high) were revealed. However, all these mutations were found in 95/95(DVG-high) as minority variants (10.8 to 46.8%) (Table 3). None of these substitutions have been reported to alter AIV pathogenicity or have any specific function. Additionally, analysis of minority variants present in the virus population showed low diversity in 95/95(DVG-low). None of the 15 polymorphic positions found in 95/95(DVG-high) was retained in 95/95(DVG-low) indicating that passages of 95/95(DVG-high) in eggs using highly diluted inoculum eliminated most of viral subpopulations (Additional file 1).

**Table 3:** **Nonsynonymous variants in virus population of 95/95(DVG-high) and 95/95(DVG-low) and their frequency in oropharyngeal swabs collected from infected turkeys**

Gene position	Reference nt	Variant nt	95/95(DVG-high) stock	95/95(DVG-low) stock	Amino acid change	Frequency in 95/95(DVG-high) turkeys	Frequency in 95/95(DVG-low) turkeys
PB1 281	T	C	0	10.2	F94S	0	0
PB1 951	G	T	10.8	100	M317I	0–77.8	100
PA 182	T	C	7.0	0	I61T	11.2–100	0
PA 574	C	A	39.0	0	R192S	0–100	0
PA 1748	G	A	21.2	100	R583Q	0–12.9	100
HA 781	A	G	0	21.3	I261V	0	0–78.3
NA 1223	G	A	33.4	100	R408K	0–100	100
M 744	G	A	46.8	100	M248I	0–100	100
NS 203	T	G	25.3	0	I68S	0–100	0

The consensus sequence of 95/95(DVG-high) was used as a reference.

*nt* nucleotide.

### Clinical outcome of infection in SPF chickens

No clinical signs or mortality were observed in chickens infected with 95/95(DVG-high) or 95/95(DVG-low). Shedding and seroconversion were noted only in directly inoculated chickens in both groups, indicating a lack of transmission to direct and indirect contact birds (Table 4). Chickens inoculated with 95/95(DVG-high) shed the virus until 7 dpi and in group inoculated with 95/95(DVG-low) shedding was observed until 14 dpi (Table 4, Additional file 1). In both groups, the viral RNA was detected almost exclusively in oropharyngeal swabs (only one cloacal swab positive in 95/95(DVG-high) group at 1 dpi).

**Table 4:** **Shedding in chickens inoculated with and exposed to 95/95(DVG-high) and 95/95(DVG-low) virus**

Dpi	95/95(DVG-high)						95/95(DVG-low)					
	Inoculated		Direct contact		Indirect contact		Inoculated		Direct contact		Indirect contact	
	OP	CL	OP	CL	OP	CL	OP	CL	OP	CL	OP	CL
1	5/5	1/5	nt	nt	nt	nt	4/5	0/5	nt	nt	nt	nt
3	4/5	0/5	0/5	0/5	0/5	0/5	4/5	0/5	0/5	0/5	0/5	0/5
5	5/5	0/5	0/5	0/5	0/5	0/5	4/5	0/5	0/5	0/5	0/5	0/5
7	3/5	0/5	0/5	0/5	0/5	0/5	4/5	0/5	0/5	0/5	0/5	0/5
10	0/5	0/5	0/5	0/5	0/5	0/5	1/5	0/5	0/5	0/5	0/5	0/5
14	0/5	0/5	0/5	0/5	0/5	0/5	1/5	0/5	0/5	0/5	0/5	0/5

The number of positive samples per the number of samples tested is indicated.

*OP* oropharyngeal swab, *CL* cloacal swab, *nt* not tested.

### Clinical outcome of infection in turkeys

**Figure 4:**
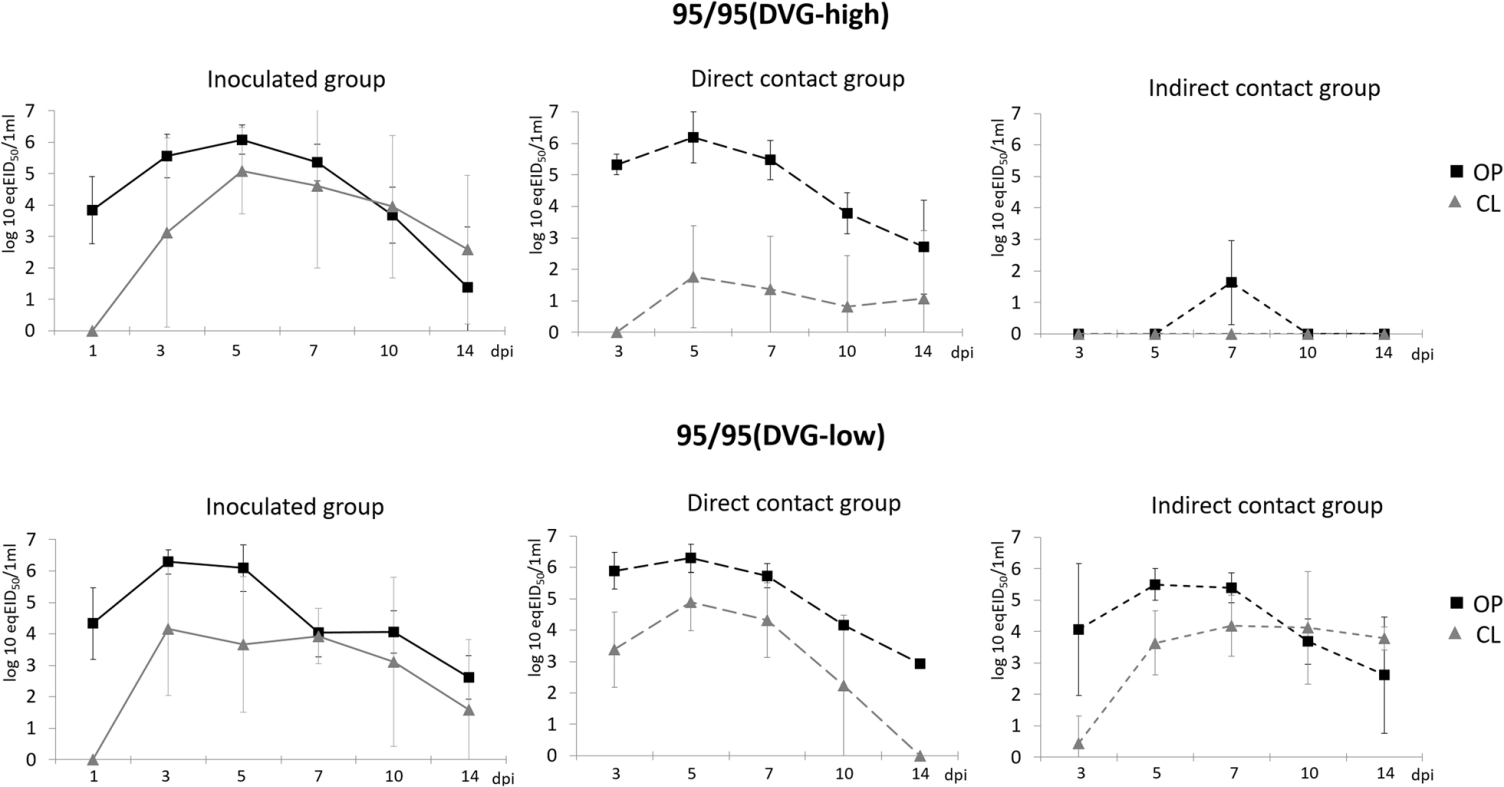
**Level of shedding in turkeys inoculated with 95/95(DVG-high) or 95/95(DVG-low) virus inoculum and in birds placed in direct or indirect contact.**

Turkeys inoculated with 95/95(DVG-high) showed slight lethargy and no mortality was observed. Respiratory and cloacal shedding was noted until 14 dpi (Table 5, Figure 4). HI titres ranged from 16 to 256. The virus was transmitted effectively to direct contact birds, as evidenced by high level of shedding (Figure 4) and seroconversion (HI titres 32–128) (Additional file 1). Poor transmission to indirect contact turkeys was observed as positive results of RT-qPCR were found only for three oropharyngeal swabs collected at 7 dpi showing low levels of viral RNA (Table 5, Figure 4) and all serum samples were negative in HI test. In contrast, a severe clinical outcome was observed in group infected with 95/95(DVG-low). First clinical signs appeared in inoculated birds at 5 dpi and from 6 dpi, clinical signs were observed also in both contact groups. Turkeys demonstrated lethargy, reluctance to move, ruffled feathers, reduced feed and water intake, dyspnoea, nasal discharge, conjunctivitis and oedema of infraorbital sinuses. A total of 9 birds died between 7 and 12 dpi, including 3 inoculated turkeys, 4 direct and 2 indirect contact birds. At necropsy, oedema and congestion of kidneys were observed in most dead turkeys. Congestion of lungs, small intestine, duodenum, pancreas and spleen were also noted in some birds. Respiratory and cloacal shedding was observed in all turkeys, indicating efficient transmission to both direct and indirect contact groups (Table 5, Figure 4). This observation was confirmed by seroconversion in all birds that survived until the end of the experiment (HI titres ranging from 16 to 256) (Additional file 1). There were no statistically significant differences in the amounts of viral RNA between 95/95(DVG-low)- and 95/95(DVG-high)-inoculated turkeys. Respiratory shedding in 95/95(DVG-low) direct contact group began at 2 dpi and continued until 14 dpi with the level of viral RNA similar to that in turkeys exposed to 95/95(DVG-high) (Table 5, Figure 4). However, differences in the patterns of cloacal shedding were observed as it was noted earlier than in 95/95(DVG-high) direct contact group. In addition, higher loads of viral RNA were found in 95/95(DVG-low) direct contact group at 3 dpi (*p* < 0.01) and 5 dpi (*p* < 0.05). The most prominent differences in the duration and intensity of shedding were noted for indirect contact groups. Both oral and cloacal shedding in 95/95(DVG-low) indirect contact turkeys was observed as soon as at 3 dpi and continued until 14 dpi (Table 5, Figure 4). The differences in the level of viral RNA were observed for oropharyngeal swabs at 3, 5, 7 and 10 dpi (*p* < 0.05) and for cloacal swabs at 5, 7 and 10 dpi (*p* < 0.01).

**Table 5:** **Shedding in turkeys inoculated with and exposed to 95/95(DVG-high) and 95/95(DVG-low) virus**

Dpi	95/95(DVG-high)						95/95(DVG-low)					
	Inoculated		Direct contact		Indirect contact		Inoculated		Direct contact		Indirect contact	
	OP	CL	OP	CL	OP	CL	OP	CL	OP	CL	OP	CL
1	5/5	0/5	nt	nt	nt	nt	5/5	0/5	nt	nt	nt	nt
3	5/5	3/5	5/5	0/5	0/5	0/5	5/5	5/5	5/5	4/5	4/5	1/5
5	5/5	5/5	5/5	3/5	0/5	0/5	5/5	4/5	5/5	5/5	5/5	5/5
7	5/5	4/5	5/5	2/5	3/5	0/5	4/4	4/4	4/4	4/4	5/5	5/5
10	5/5	4/5	5/5	1/5	0/5	0/5	3/3	2/3	2/2	1/2	4/4	4/4
14	2/5	3/5	4/5	1/5	0/5	0/5	2/2	1/2	1/1	0/1	2/3	3/3

The number of positive samples per the number of samples tested is indicated.

*O*P oropharyngeal swab, *CL* cloacal swab, *nt* not tested.

### Analysis of virus sequences in swabs

Due to the differences in the clinical manifestation of infection with 95/95(DVG-high) and 95/95(DVG-low) in turkeys, a possibility of transformation into highly pathogenic form was taken into consideration. To verify this possibility, samples collected at the end of the experiment were subjected to HA cleavage site sequencing. All tested samples showed typical monobasic HACS (PEIPKGR*GLF) indicating low pathogenic phenotype. Additionally, sequences generated in deep sequencing were analysed to reveal any mutation that could have an effect on the pathobiological outcome. The presence of nonsynonymous mutations that differentiated both virus stocks and those newly emerged was investigated in swabs from 5 dpi. Sequences of viruses from 95/95(DVG-low) group reflected the dominant population in the virus inoculum, i.e. in most birds there were no differences at the consensus level. Seven birds showed a nonsynonymous mutation at the HA protein (I261V) (frequency of 5.5–78.3%) which was already present as a minority variant in the virus stock (Table 3, Additional file 1). The 95/95(DVG-high) group showed high variation in the frequency of the nonsynonymous mutations between birds (Table 3, Additional file 1) indicating a lack of particular selection pattern.

## Discussion

Pathogenicity of avian influenza viruses depends on host- and virus-related factors. The traditional classification into low and highly pathogenic AIV is based on the result of intravenous inoculation of the virus into chickens. However, gallinaceous poultry are considered more susceptible to AIV infection than waterfowl [35] and clinical course of LPAIV infection can be sometimes more severe than HPAIV infection in ducks or geese [36, 37]. Moreover, virus-specific factors can also influence pathogenicity, a feature that has been well described in Gs/GD H5 HPAIV lineage viruses, even closely related ones [38]. Additionally, pathogenicity experiments that are not performed in specific pathogen free birds should also take into account the subclinical presence of other pathogens that may exacerbate the infection. So far, the possible variation in clinical outcome following infection with the same virus strain in the same species of birds has gained little attention even though the potential implications caused by the interference of defective viral particles on the replication of fully infectious particles have been known for a few decades [5]. In our study we investigated the pathogenicity and transmissibility of a turkey-origin low pathogenic AIV H7N7 strain with a high and low load of DVGs in SPF chickens and in AIV-negative turkeys. Since the turkeys used in the study were not obtained from a specific pathogen free flock, a number of tests were carried out prior to the experiment to exclude the presence of potential subclinical infections with the most common turkey pathogens. Infected birds received the same infectious dose of the virus but with different amount of DVGs. The semiquantitative analysis of defective particles was done by a combination of RT-PCR, real time RT-PCR and whole genome sequencing and indicated significantly higher amount of truncated gene segments in 95/95(DVG-high). Consequently, the infectious titre was higher in 95/95(DVG-low). Sequencing of defective segments showed patterns similar to those described previously in influenza viruses, i.e. they were generated mostly from polymerase complex genes by deletion of a large middle fragment while retaining 3′ and 5′ packaging signals [18, 39].

No significant differences were observed in chickens as inoculated birds remained healthy, shed moderate amounts of the virus without transmission to direct and indirect contact chickens. The longer shedding duration of 95/95(DVG-low) than 95/95(DVG-high) might indicate slightly better replication efficiency of 95/95(DVG-low) but requires further investigations.

On the other hand, the experiment in turkeys showed striking differences in pathogenicity and transmissibility between 95/95(DVG-high) and 95/95(DVG-low) AIVs. Infection of turkeys with the 95/95(DVG-high) virus stock induced mild clinical signs with no mortality and resulted in transmission only to birds placed in direct contact. In contrast, severe respiratory and systemic disease was noted in turkeys inoculated with 95/95(DVG-low) AIV as well as in direct and indirect contact turkeys followed by mortality in all groups (cumulative mortality of 60%). The possibility that the severe clinical outcome observed in 95/95(DVG-low)-infected turkeys had been caused by the transition of the virus into highly pathogenic form was ruled out by sequencing of the postpassage virus and demonstration of the typical LPAIV cleavage site. Additionally, the comparison of the whole-genome sequence of the inoculum and virus excreted by birds showed no difference at the consensus level. Since the amino-acid sequences of dominant populations in both virus stocks differed at four sites, analysis of polymorphisms at these positions in virus populations from swabs was also performed. Turkeys infected with 95/95(DVG-high) showed high variability in the frequency of the analysed variants, while no polymorphisms were found in turkeys infected with 95/95(DVG-low) which reflected the homogeneity of the virus inoculum. The maintenance of both variants at each position in turkeys infected with 95/95(DVG-high) and high between-host diversity in terms of frequency suggests that none of these mutations conferred any specific advantage for the virus replication efficiency or transmissibility. This allows to draw the conclusion that the difference in the amount of defective particles was the factor responsible for the observed disparities in the pathobiology of 95/95(DVG-high) and 95/95(DVG-low).

There are two possible explanations of the significant pathobiological differences between LPAIV with high and low DIPs load. Firstly, the production of incomplete particles at the expense of fully infectious particles led to the attenuation of clinical outcome, decline in mortality rate and reduction in transmission efficiency. There were no statistically significant differences in the amount of viral RNA between inoculated birds but the earlier onset and higher level of cloacal shedding in the 95/95(DVG-low) direct contact turkeys and high oral and cloacal shedding in the indirect contact group suggest that higher amounts of fully infectious particles were transmitted to turkeys exposed to 95/95(DVG-low) enabling more efficient and faster dissemination of virus within the host. This hypothesis is supported also by the gross lesions and high load of viral RNA found in kidneys (data not shown) indicating that higher amounts of infectious particles (or the lack of the interfering activity of DIPs) enabled systemic spread of the virus. The second explanation is that DIPs trigger innate immune response at the early stage of infection. It was shown recently that defective viral genomes of human respiratory syncytial virus stimulated the antiviral response in mice and humans [40]. It is also possible that the observed outcome is a combined effect of early antiviral response triggered by DIPs accompanied by the interference with the generation of viral particles with complete infectious capacities. The protective effect of DIPs in influenza infection has been shown in mice and ferrets [41–44] and a relationship between the severity of infection and amount of DIPs was also identified in humans [18]. Differences in pathogenicity that could be attributed to abundance of DIPs were also found for H5N2 AIV [16, 23]. However, the role of DIPs in natural infections of avian hosts is unknown and their presence in field samples has been rarely reported [45]. It is possible that under field conditions there are fluctuations in the proportions between quantities of complete and defective particles at flock level, leading to the alternate phases of suppression and exacerbation of clinical outcome. This hypothesis needs further verification in an experiment with a longer chain of subsequent transmissions and analysis of viral populations derived from each passage but such phenomenon could be perceived as advantageous from the perspective of survival and subsequent spread of the virus.

In conclusion, to the best of our knowledge this is the first report investigating the role of AIV containing defective genomes in the modulation of disease outcome in birds under experimental conditions. Significant differences observed in turkeys can result from either the suppressive effect of DIPs on the production of functional viral particles capable of causing disease and/or the early stimulation of innate antiviral response. The results can also have implications for the interpretation of virulence assessment results routinely conducted for AIV field isolates.

## Supplementary information

**Additional file 1.** Frequency of variants found in viral populations of virus stocks and swabs collected from infected turkeys and results of quantitative analysis of virus shedding.

## Data Availability

The datasets generated in the current study are included in the article or in Additional file, or are available from the corresponding author on reasonable request.

## Abbreviations

AIV: avian influenza virus
DIP: defective interering particle
dpi: day post infection
DVG: defective viral genome
EID_50_: 50% embryo infectious dose
eqEID_50_: equivalent of EID_50_
HA: hemagglutination
HACS: hemagglutinin cleavage site
HI: hemagglutination inhibition
HPAIV: highly pathogenic avian influenza virus
LPAIV: low pathogenic avian influenza virus
nt: nucleotide
RT-qPCR: quantitative real time RT-PCR
SPF: specific pathogen free
